# The flavonoid-rich Quzhou Fructus Aurantii extract modulates gut microbiota and prevents obesity in high-fat diet-fed mice

**DOI:** 10.1038/s41387-019-0097-6

**Published:** 2019-10-23

**Authors:** Yong-feng Bai, Si-wei Wang, Xiao-xiao Wang, Yuan-yuan Weng, Xue-yu Fan, Hao Sheng, Xin-tian Zhu, Li-jun Lou, Feng Zhang

**Affiliations:** 1 Department of Clinical Laboratory, Quzhou People’s Hospital, 324000 Quzhou, China; 2 Department of Core Facility, Quzhou People’s Hospital, 324000 Quzhou, China; 3 Department of Pharmacy, Quzhou People’s Hospital, 324000 Quzhou, China; 4 Department of Drug Analysis Center, Quzhou Institute for Food and Drug Control, 324000 Quzhou, China; 5 Medical School, Zhejiang University, 310058 Hangzhou, China; 6 Wenzhou Medical University, 325000 Wenzhou, China

**Keywords:** Type 2 diabetes, Bacteria, Nutrition

## Abstract

**Background:**

Flavonoids are reported to modulate the composition of gut microbiota, which play an important role in preventing obesity and associated metabolic diseases. In this study, we investigated the effect of Total Flavonoids of Quzhou Fructus Aurantii Extract (TFQ) on gut microbial community in mice fed with a high-fat diet (HFD).

**Methods:**

C57BL/6J mice were fed with either a chow diet or HFD with or without oral gavage of TFQ (300 mg/kg/day) for 12 weeks.

**Results:**

Our data indicate TFQ significantly reduced obesity, inflammatio,n and liver steatosis. TFQ elevates the expression of tight junction proteins and reduces metabolic endotoxemia. In addition, TFQ treatment reverses HFD-induced gut dysbiosis, as indicated by the reduction of *Firmicutes* to *Bacteroidetes* ratio, the increase of genera *Akkermansia* and *Alistipes*, and the decrease of genera *Dubosiella*, *Faecalibaculum*, and *Lactobacillus*.

**Conclusion:**

These findings support a prebiotic role of TFQ as a dietary supplement for the intervention of gut dysbiosis and obesity-related metabolic disorders.

## Introduction

Obesity is a growing public health concern which strongly influences the quality of human life^1^. Growing evidence indicates that chronic inflammation caused by obesity is an independent risk factor for chronic diseases such as insulin resistance, nonalcoholic fatty liver disease and inflammatory bowel disease, and it significantly increases the morbidity and mortality of these diseases^2–4^.

In recent years, with the advances in human microbiota research, accumulating evidence shows that gut microbiota dysbiosis is tightly associated with obesity^5,6^. Gut microbiota is involved in regulating various physiological metabolic processes, which are vital to the digestion, nutrition, metabolism, and immunity of our bodies^7^. The dysbiosis of gut microbiota, which is mostly manifested as a decrease in bacterial richness and diversity, may induce low-grade inflammation, leading to metabolic diseases such as obesity^8,9^. Studies have shown that high-fat diet-induced obesity disrupts mucosal barrier integrity and leaks bacterial lipopolysaccharide (LPS) from the intestinal lumen into bloodstream, leading to metabolic endotoxemia and body weight gain^7,10^. Therefore, modulation of gut microbiota may represent a novel therapeutic approach for obesity and metabolic syndrome treatment.

Flavonoids are important dietary supplements with anti-obesity and anti-diabetic properties^11^. The clinical and experimental studies have provided compelling evidence that flavonoids intake aids in preventing obesity and type 2 diabetes^12,13^. Flavonoids can be transformed by gut microbes into metabolites with increased or decreased biological activities^14^. Meanwhile, flavonoids such as hesperidin^15^, quercetin^16^, and *Cyclocarya paliurus* flavonoid^17^, have been reported to modulate the composition of gut microbiota by increasing probiotics and reducing pathogens, which play an important role in preventing obesity and associated metabolic diseases. The complex interaction between flavonoids and gut microbiota is essential for the pharmacological activities of these natural products^18^.

Quzhou Fructus Aurantii is a dried unripe fruit of Rutaceae Citrus changshan-huyou, which is recorded in the “Zhejiang Traditional Chinese Medicine Processing Norms (2015)”^19^. Our previous study has shown that the main components of Quzhou Fructus Aurantii are naringin, narirutin, hesperidin and neohesperidin, which constitute an important sort of flavonoids^20^. In the practice of traditional Chinese medicine, Quzhou Fructus Aurantii is often used in the treatment of gastrointestinal diseases. However, the effect of Quzhou Fructus Aurantii on gut microbiota has not been reported. In this study, we investigated the influence of the Quzhou Fructus Aurantii extract, 60–70% of which consisting of flavonoids, on gut microbiota in a high-fat diet-fed mice model. Our results, for the first time, demonstrate that the flavonoid-rich Quzhou Fructus Aurantii extract ameliorates fatty liver, insulin resistance and intestinal inflammation through the modification of gut microbiota at least in part.

## Materials and methods

### The total flavonoids of Quzhou Fructus Aurantii Extract (TFQ)

Quzhou Fructus Aurantii was purchased from Quzhou Nankong Chinese Medicine Co., Ltd. The dried Quzhou Fructus Aurantii was crushed and extracted three times with ethanol/water (80:20, v/v) at 60 °C for 2 h each time. Then the extract was filtered and freeze-dried. The flavonoid composition in Quzhou Fructus Aurantii extract was determined by high performance liquid chromatography (HPLC). The characterizations of its flavonoids are shown in Table 1.

**Table 1:** Chemical characterization of Quzhou Fructus Aurantii extract

Flavonoid composition	Content (%)	Daily intake (mg/kg body weight)
Narirutin	4.57 ± 0.06	13.71 ± 0.18
Naringin	41.00 ± 0.35	122.91 ± 1.19
Hesperidin	2.63 ± 0.08	7.89 ± 0.24
Neohesperidin	20.61 ± 0.12	61.83 ± 0.36

### Animal experiments

Animal experiments were approved and performed in accordance with the guidelines of Committee on the Ethics of Animal Experiments of Zhejiang University of Traditional Chinese Medicine, China. Thirty-six 8-week-old C57BL/6J male mice (Shanghai Laboratory Animal Center) were housed under standard pathogen-free conditions with controlled light conditions (a light–dark cycle 12 h) and food and water ad libitum. After 2 weeks of acclimation, 36 mice were randomly distributed into three groups of 12 mice (4 mice per cage): (a) Chow group, which fed a chow diet; (b) high- fat diet (HFD) group, which fed a HFD with 60 kcal% fat (Research diet D12492, Research Diet, NJ); (c) TFQ group, which fed HFD and daily dose of 300 mg/kg TFQ by gavage for 12 weeks. Body weight gain and food intake were assessed once a week. At the end of the experiment, the samples of colonic contents for microbiological examination were collected.

### Glucose homeostasis

For insulin tolerance tests (ITT), at week 7, mice were 6 h fasted and injected i.p. with insulin (0.75 U/kg body weight). An automatic glucometer (OneTouch Ultra, Lifescan, Milpitas, CA) measured tail vein blood glucose at 0, 30, 60, 90, and 120 min after injection. Oral glucose tolerance tests (OGTT) were performed after 12 h fasts at the end of week 8. The glucose (2 g/kg body weight) was delivered by gastric gavage. The glucose levels were monitored at 0, 15, 30, 60, 90, and 120 min as before mentioned.

### Biochemical testing

Serum alanine transaminase (ALT), aspartate transaminase (AST), total cholesterol (TC), triglyceride (TG), high density lipoprotein cholesterol (HDL-C), low density lipoprotein cholesterol (LDL-C) and non-esterified fatty acids (NEFA) were measured according to the manufacturer’s instruction (DiaSys Diagnostic Systems, Shanghai, China). Serum insulin and lipopolysaccharides (LPS) concentration, was tested by enzyme-linked immunosorbent assay (ELISA) kits according to the manufacturer’s instructions (MEIMIAN, China). The homeostasis model assessment of insulin resistance (HOMA-IR) index was calculated according to the formula: fasting insulin (mU/ml) × fasting glucose (mmol/L)/22.5. Liver triglyceride (TG), as well as cholesterol (TC) content was assessed using enzymatic reactions with commercial kits (Dongou Diagnostics Co., LTD, Zhejiang, China).

### Pathological staining

Liver and epididymal white adipose tissues were carefully collected for hematoxylin-eosin (H&E) staining. Non-alcoholic fatty liver disease (NAFLD) activity score is the unweighted sum of steatosis, lobular inflammation and hepatocellular ballooning score according to the Brunt system^21^.

### Quantitative real-time PCR (qPCR)

Total RNA was isolated using TRIzol reagent (Tiangen Biotech, China) according to the manufacturer’s instructions. RNA concentration was evaluated by absorbance at 260 and 280 nm using Nano-100 microscope spectrophotometer (Allsheng Instruments, China). Complementary DNA (cDNA) synthesis was performed using reverse transcriptase kits (Thermo Fisher Scientific, Waltham, MA) following the manufacturer’s instructions. Real-time PCR was performed using SYBR Green (Sangon Biotech, China) on LightCycler 480 instrument (Roche, Basel, Switzerland). The relative gene quantities were calculated by the 2^−ΔΔCt^ method in comparison with the expression levels of *GAPDH*. The primers are listed in Table 2.

**Table 2:** The primers used in this study for real time PCR

Description	Sense primer (5′ → 3′)	Antisense primer (5′ → 3′)
*Cldn3*	CAGGGGCAGTCTCTGTGCGAG	GCCGCTGGACCTGGGAATCAAC
*Ocln*	ATGTCCGGCCGATGCTCTC	TTTGGCTGCTCTTGGGTCTGTAT
*GAPDH*	TGAGGCCGGTGCTGAGTATGT	CAGTCTTCTGGGTGGCAGTGAT

### Western blotting

Western blotting was performed as described previously^22,23^. Protein from each colon sample was extracted with lysis buffer (50 mM Tris-HCl, pH 7.4, 2 mM EDTA, 150 mM NaCl, 0.1% sodium dodecyl sulfate, and 1% NP-40), supplemented with protease inhibitor cocktail and phosphatase inhibitor cocktail (1 mM sodium orthovanadate, 5 mM sodium fluoride, 3 mM β-glycerophosphate, and 4 mM sodium tartrate) at 4 °C. Then the extracts were centrifuged at 4 °C for 10 min at 16,000 × *g* and separated supernatant for western blotting analyses. The equal amounts of proteins boiled for 5 min, then separated by 10–15% SDS-PAGE and transferred to PVDF membranes. The membranes were blocked in 1% casein for detection of proteins, incubated overnight at 4 °C in primary antibodies and visualized with secondary HRP-conjugated antibodies. Immune complexes were detected by the Tanon 4200SF system from Tanon Biotechnology (Shanghai, China). Band intensity was quantified using ImageJ software (U.S. National Institutes of Health, Bethesda, MD). The primary antibodies used in this study were anti-phospho-NF-κB p65 (1:1000, #3033, Cell Signaling Technology), anti-phospho-IKKα/β (1:1000, #2697, Cell Signaling Technology), anti-TNF-α (1:500, ab183218, Abcam), anti-COX-2 (1:500, ab179800, Abcam), and anti-β-actin (1:3000, A1978, Millipore Sigma).

### Gut microbiota analysis

The genomic DNA of colonic content was extracted with modified CTAB method. V3, V4 hypervariable regions of microbial 16S rRNA genes were amplified using the MetaVxTM Library Preparation kit (GENEWIZ, Inc., South Plainfield, NJ.USA). Sequencing was accomplished on the Illumina HiSeq platform by GENEWIZ, Inc. (Suzhou, China) using a method described previously^24^. Sequences were clustered into operational taxonomic units (OTUs) using a 97% identity cutoff. The Ribosomal Database Project (RDP) classifier uses a Bayesian approach to assign 16S rRNA sequences into different taxonomic levels. MicrobiomeAnalyst^25^, a web-based tool, was used to calculate alpha diversity analysis, principal coordinate analysis (Bray-Curtis distances) and comparative analysis of microbiome data. Biomarker discovery using Linear Discriminant Analysis Effect Size (LEfSe) was performed online (http://huttenhower.sph.harvard.edu/galaxy).

### Statistical analysis

Data are graphed as mean and error bars show standard deviation (S.D.) unless otherwise stated. All data were analyzed using one way analysis of variance (ANOVA) by SPSS software (version 20.0) or MicrobiomeAnalyst program. Differences were considered to be statistically significant at *P* < 0.05 and highly significant at *P* < 0.01.

## Results

### TFQ ameliorates obesity and fatty liver and improves insulin resistance in HFD-fed mice

**Fig. 1:**
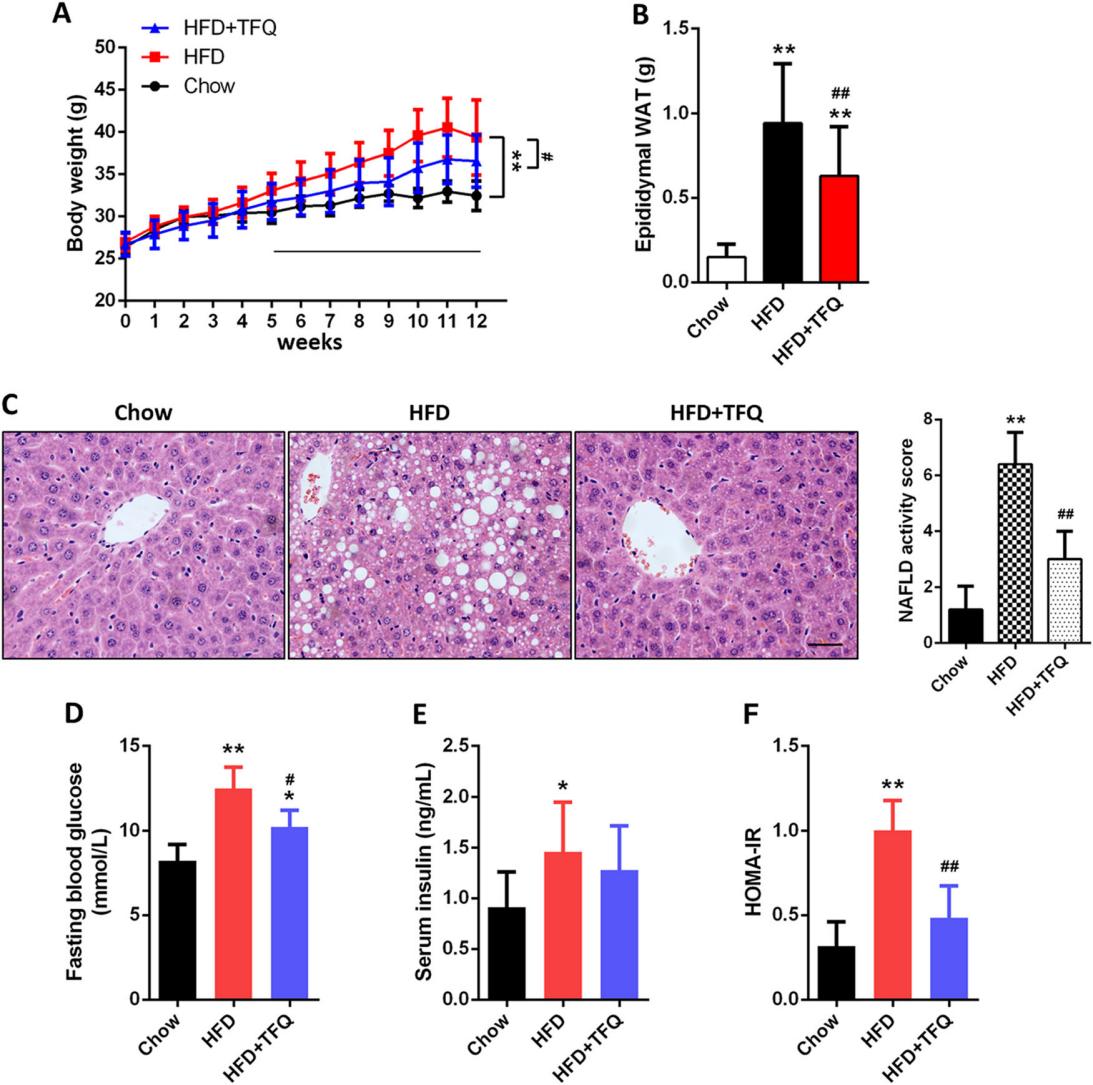
The effect of TFQ on obesity, fatty liver and insulin resistance in HFD-fed mice. C57BL/6J mice were fed either a chow or a high-fat diet (HFD) for 12 weeks. Mice were treated with daily oral doses of TFQ (300 mg/kg). Water was gavaged as control. **a** Body weight curve (*n* = 12). **b** Epididymal white adipose tissue (WAT) weight (*n* = 12). **c** Left, the representative images of H&E staining in liver from each group. Scale bar, 300 μm. Right, NAFLD activity scores in each group (*n* = 5). **d** Fasting blood glucose level in last week (*n* = 12). **e** Serum insulin level (*n* = 12). **f** Homeostasis model assessment of insulin resistance (HOMA-IR) index (*n* = 12). Data were expressed as the mean ± SD. *p < 0.05, **p < 0.01, vs. chow group; ^#^*p* < 0.05, ^##^*p* < 0.01, vs. HFD group

As depicted in Fig. 1a and S1A, compared to chow-fed mice, the mice gained significantly more body weight after 5-week HFD feeding. HFD feeding also resulted in marked increase of the epididymal white adipose tissue (WAT) mass (Fig. 1b and S1B). TFQ gavage at a dose of 300 mg/kg significantly reduced HFD-induced weight gain of the whole body and the epididymal WAT (Fig. 1a, b). The H&E staining of epididymal WAT revealed that the administration of TFQ prominently attenuated HFD-induced adipocyte hypertrophy and hyperplasia. (Fig. S1B).

After 12-week HFD feeding, mice developed obvious hyperlipidemia and fatty liver, reflected by elevated TC and TG levels in serum and liver tissue. As shown in Fig. S2A and S2B, the serum TC and TG levels were evidently increased in HFD-fed mice compared to normal chow counterparts. These indicators were significantly decreased after treatment with TFQ. TFQ also reduced HFD-induced serum NEFA and LDL-C, but had no effect on HDL-C level in these mice (Fig. S2C-E). Moreover, administration of HFD-fed mice with TFQ decreased hepatic TG accumulation and liver weight, concomitantly reducing serum ALT and AST, while had no effect on hepatic TC (Fig. S3). The results of H&E staining showed that the HFD induced obvious hepatic lobular disorders, hepatic steatosis and hepatocellular ballooning with inflammatory cell infiltrations in mice. TFQ administration ameliorated all these liver histopathological changes and decreased the NAFLD activity scores (Fig. 1c).

Insulin resistance is one of the commonest pathological features in HFD fed mice^26^. As expected, HFD feeding markedly elevated fasting blood glucose (FBG) (Fig. 1d) and insulin levels (Fig. 1e). Although TFQ treatment did not significantly lower insulinemia in HFD-fed mice, it obviously decreased FBG and HOMA-IR levels (Fig. 1d and f), suggesting that TFQ ameliorated insulin sensitivity in HFD-fed mice. We next conducted oral glucose-tolerance tests (OGTT) and insulin-tolerance tests (ITT) to further assess the effect of TFQ on glucose homeostasis and insulin sensitivity. The administration of TFQ lessened values of OGTT in HFD-fed mice (Fig. S4A). In addition, TFQ treated mice displayed a downward trend (*P* = 0.0632) in AUC-ITT compared to HFD-fed model group (Fig. S4B).

### TFQ suppresses serum LPS level and intestinal inflammation in HFD-fed mice

**Fig. 2:**
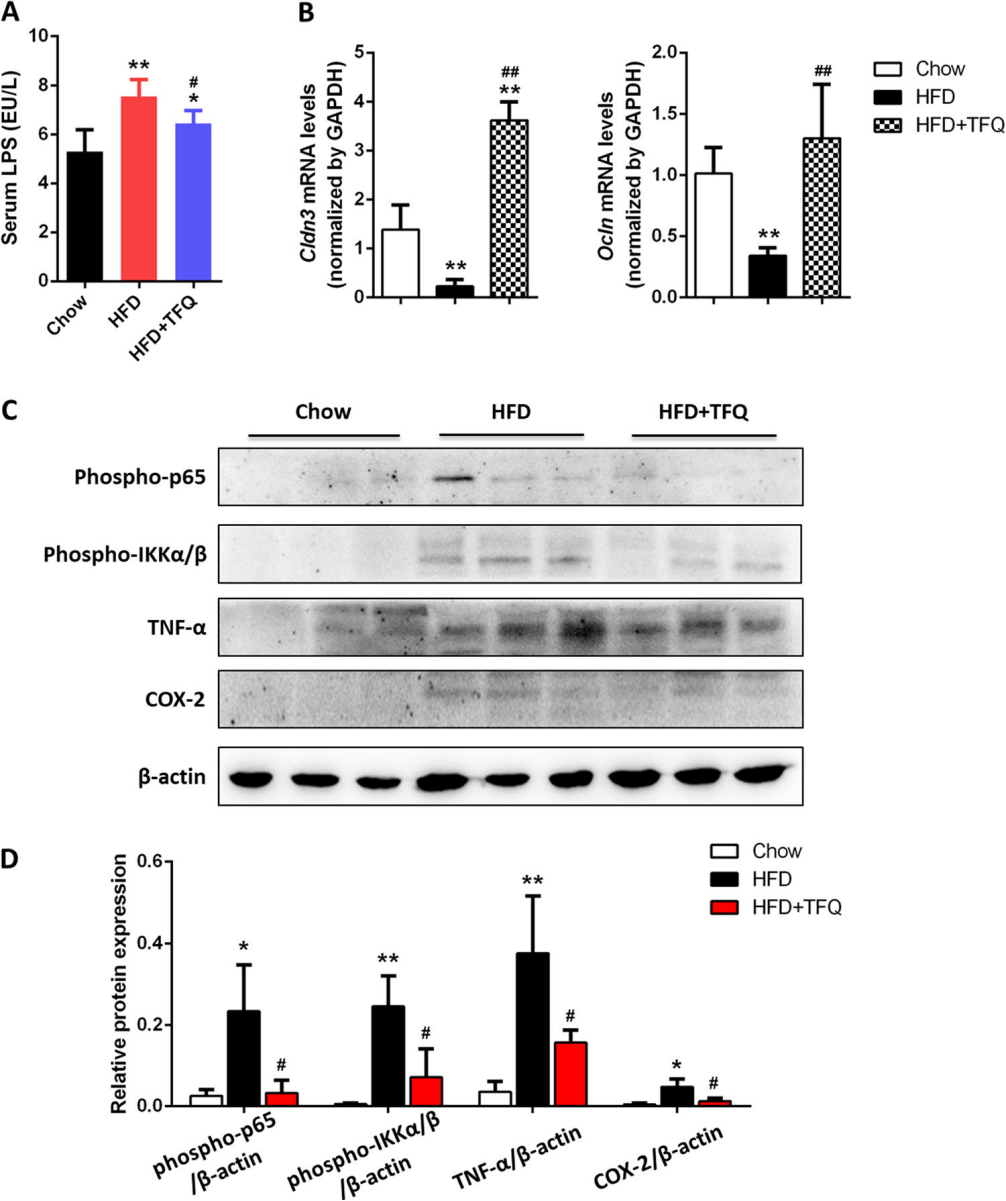
The effect of TFQ on intestinal inflammation in HFD-fed mice. **a** Serum LPS level in mice. Data were expressed as the mean ± SD (*n* = 12). **b** The mRNA expression of *Cldn3* and *Ocln* were assessed by RT-PCR. **c**The protein levels of phospho-p65, phospho-IKKα/β, TNF-α, and COX-2 were assessed by western blot analysis. **d** The density of bands was normalized to β-actin. Values were expressed as mean ± SD (*n* = 3). *p < 0.05, **p < 0.01, vs. chow group; ^#^*p* < 0.05, ^##^*p* < 0.01, vs. HFD group

It has been reported that HFD could change intestinal permeability and cause low-grade intestinal inflammation through raising circulating LPS level^27^. As shown in Fig. 2a, 12 weeks of HFD feeding significantly raised serum LPS level, which was reduced by TFQ administration. The expression of *Cldn3* and *Ocln* in colon tissues, which are major components of tight junction and play key roles in regulating gut permeability^28^, was significantly lower in HFD fed mice and remarkably recovered after TFQ treatment (Fig. 2b). To further determine the effect of TFQ on intestinal inflammation induced by HFD, we also examined the expression of intestinal inflammatory cytokines in mice. The results showed that TFQ administration markedly inhibited the phosphorylation of NF-κB p65 and IKKα/β, as well as the amount of TNF-α and COX-2 (*P* < 0.05) (Fig. 2c, d).

### TFQ modulates the overall composition of intestinal bacterial community

**Fig. 3:**
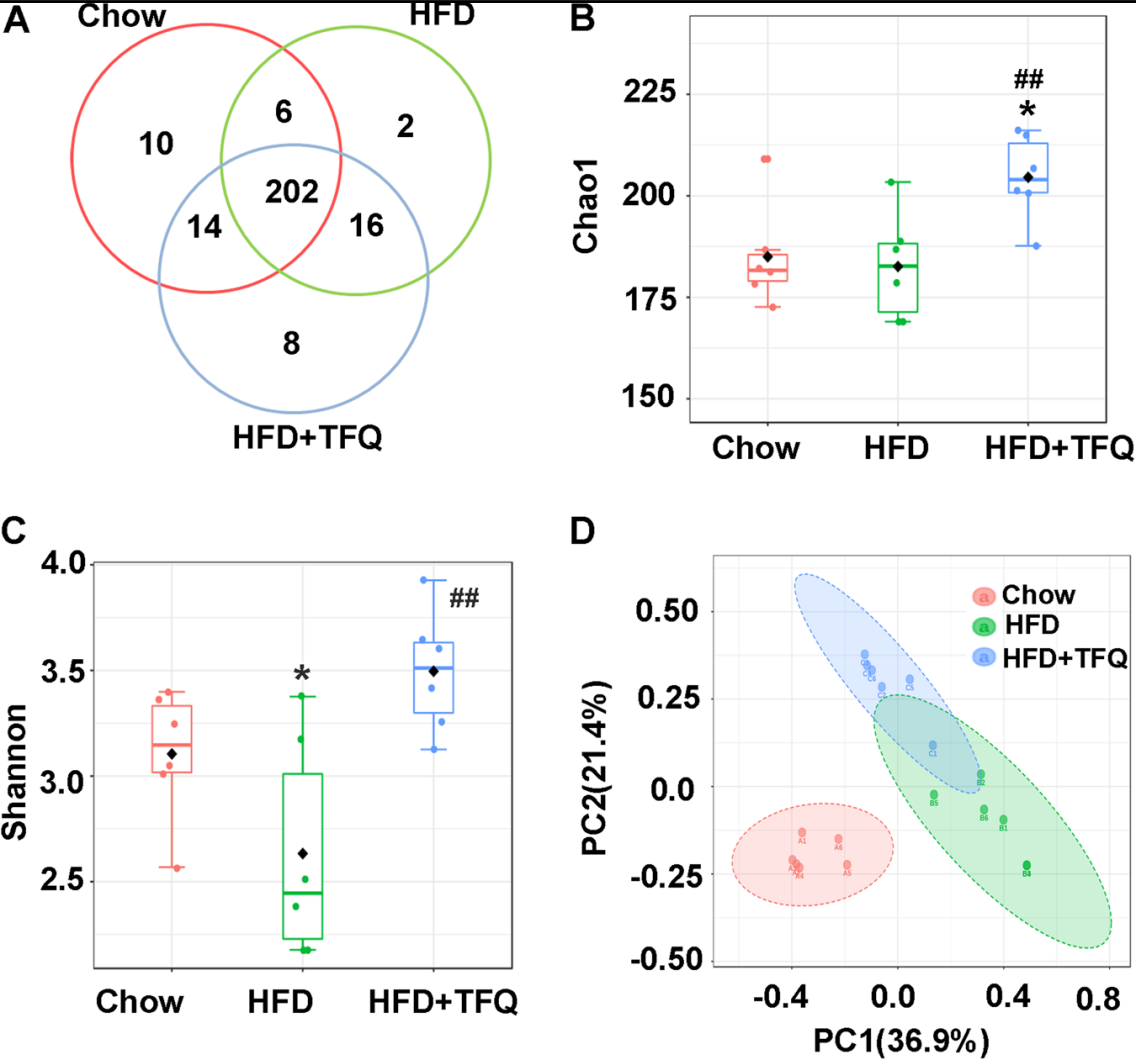
TFQ modulates the overall composition of bacterial community. **a** A Venn diagram showing shared and unique OTUs among the three groups. The Chao1 index (**b**) and the Shannon index (**c**) for alpha diversity estimation. **d** Principal-coordinate analysis (PCoA) plot showing the similarity relations among the three groups. *n* = 6 per group. *p < 0.05, **p < 0.01, vs. chow group; ^#^*p* < 0.05, ^##^*p* < 0.01, vs. HFD group

The intestinal flora is crucial in the pathogenesis of obesity and related metabolic diseases. To investigate whether TFQ regulated gut microbiota of HFD-fed mice, we performed 16S rRNA sequencing to analysis of the bacterial community structure in specimen of intestinal contents. The Venn diagram showed the numbers of OTUs detected in each group. There are 202 OTUs shared by three groups. Meanwhile, each group had unique OTUs (Fig. 3a). Treatment with TFQ significantly increased the alpha diversity of the gut microbiota as indicated by the Chao1 index and the Shannon index (Fig. 3b, c). Principal coordinate analysis (PCoA) was used to determine clustering patterns among three groups. The gut microbiota of the TFQ group was closer to the control group in PCoA plot (Fig. 3d). These results showed that TFQ treatment improved the structure of the intestinal flora.

### TFQ manipulates the specific phylotypes of the gut microbiota

**Fig. 4:**
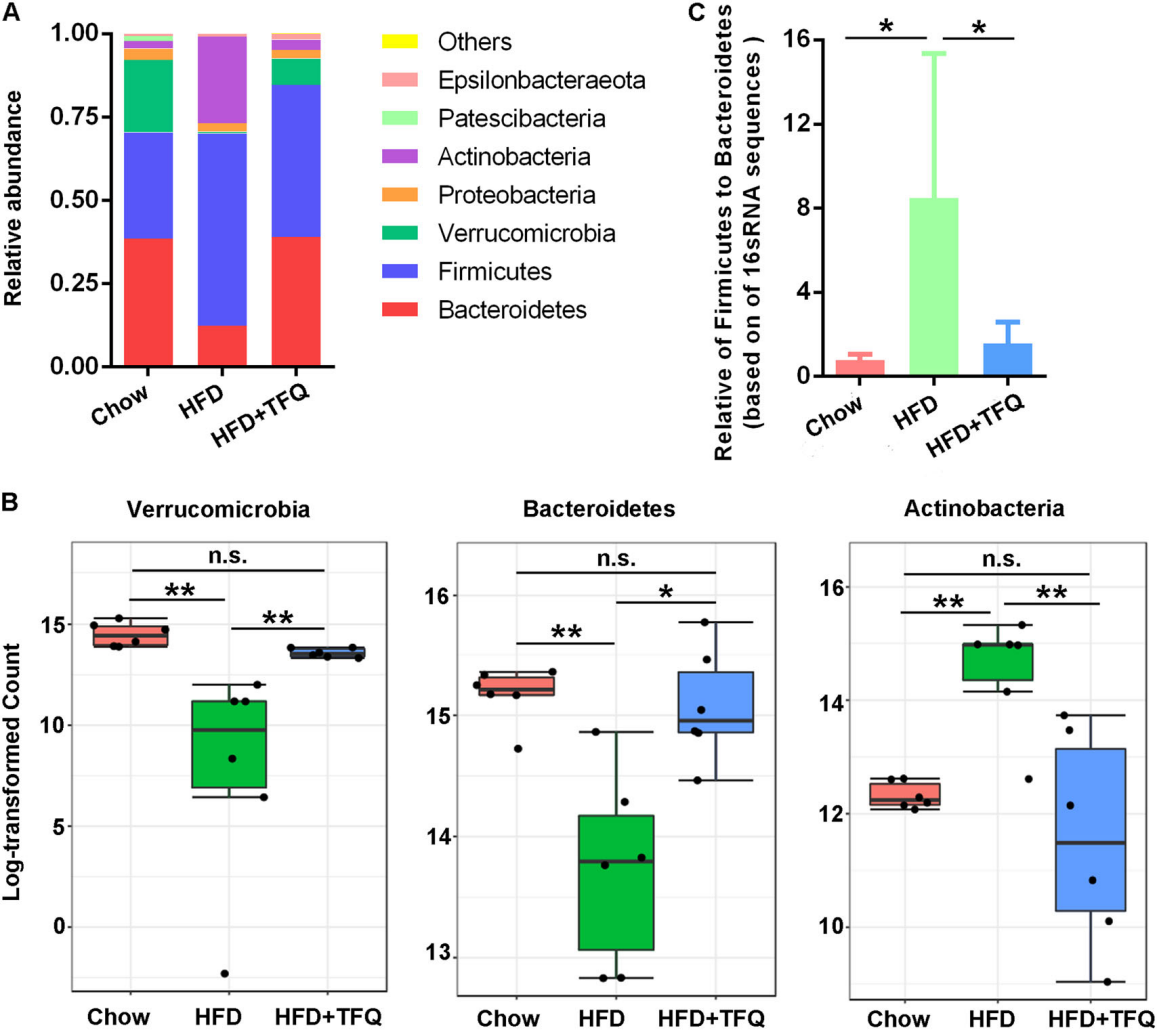
The change of the gut microbiota structure at the phylum level. **a** Histogram of the relative abundance of the dominant bacterial phyla. **b** TFQ reversed the specific bacterial phyla. **c** The ratio of Firmicutes to Bacteroidetes. Values were expressed as mean ± SD. *n* = 6 per group. *p < 0.05; **p < 0.01; n.s., no significance

The histograms illustrating the gut microbiota community structure revealed the microbial species and their relative abundance at the phylum level (Fig. 4a). The proportion of *Verrucomicrobia* and *Bacteroidetes* was markedly decreased, whereas the relative abundance of *Actinobacteria* was significantly increased in the HFD-fed mice compared to their chow-fed counterparts. In contrast, TFQ treatment corrected relative abundance of these bacterial groups (Fig. 4b). *Firmicutes* and *Bacteroidetes* are the two most abundant bacterial phyla in the intestinal tracts of experimental mice. The gut microbiota of HFD-fed mice was characterized by a dramatic increase of the *Firmicutes* to *Bacteroidetes* ratio compared with that of the chow-diet group, and this trend was also reversed by the TFQ treatment (Fig. 4c).

**Fig. 5:**
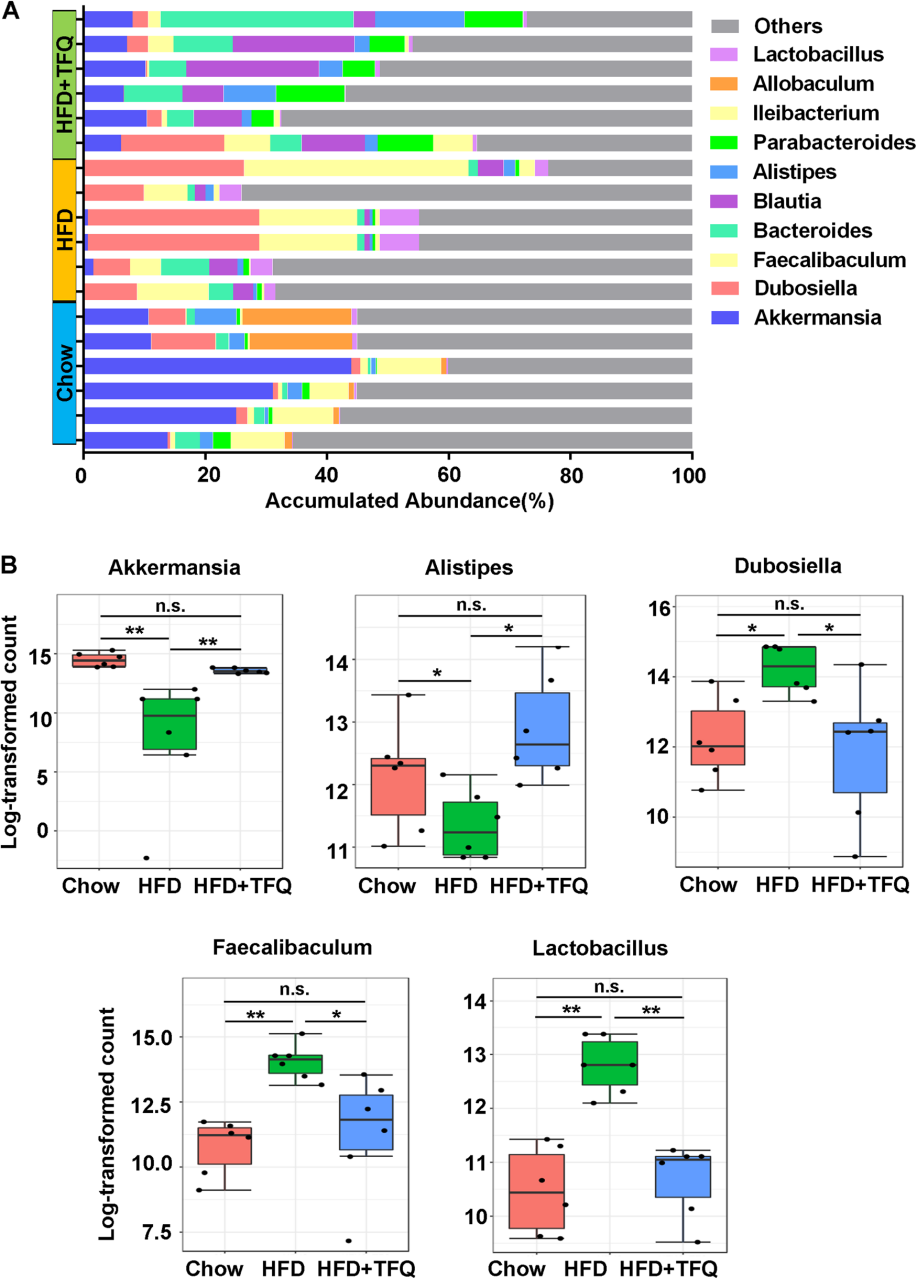
The change of the gut microbiota structure at the genus level. **a** The accumulative abundance of the top 10 genera among the different groups. **b** TFQ reversed the specific bacterial genera. *n* = 6 per group. *p < 0.05; **p < 0.01; n.s., no significance

Figure 5 displays the abundance changes of the top 10 genera among the three different groups. At the genus level, HFD feeding reduced *Akkermansia* and *Alistipes* and enlarged *Dubosiella*, *Faecallbaculum*, and *Lactobacillus*. Notably, TFQ could partly reverse these changes. These obvious alterations of individual genera after TFQ treatment not only confirmed the modulatory effect of TFQ on the gut microbiota but also implied that these genera might be key bacteria to the therapeutic effects of TFQ.

### TFQ changes taxonomic biomarkers in each group

**Fig. 6:**
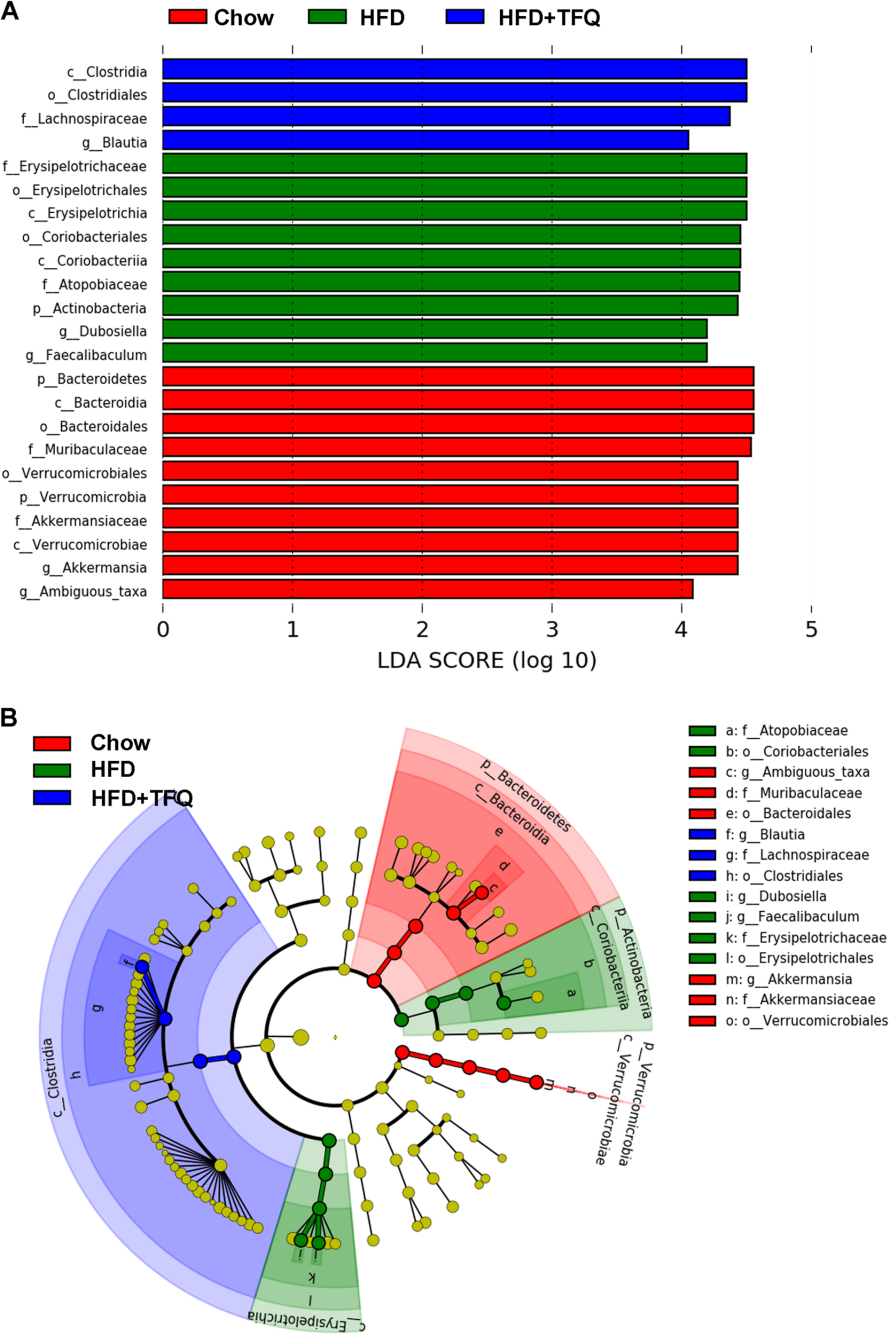
LEfSe analysis of taxonomic biomarkers of gut microbiota. **a** LEfSe analysis identified the most differentially abundant taxons. LDA score > 4 are shown. **b** Cladogram of significant changes at all taxonomic levels. The root of the cladogram represents the domain bacteria. The size of node represents the abundance of taxa. *n* = 6 per group

In addition, to identify the characteristic bacteria which were specific for each group, linear discriminant analysis (LDA) effect size (LEfSe) algorithm approach was applied. The chow group showed the most unique microbiota by a high abundance of genera *Ambiguous*-taxa (class within *Bacteroidia*) and *Akkermansia* (class within *Verrucomicrobiae*). The genera of *Faecallbaculum* and *Dubosiella* (class within *Erysipelotrichia*) and family *Atopotiaceae* (class within *Coriobacteriia*) were the dominant phylotypes that contributed to the differences between the intestinal microbiota of chow and HFD-fed mice. The abundance of genus *Blautia* (class within *Clostridia*) was higher in the TFQ group (Fig. 6).

## Discussion

It is believed that flavonoids and their metabolites could protect against obesity-related diseases^12,18^. Given that flavonoids are a sort of the main components of Quzhou Fructus Aurantii extract, we investigated the effect of TFQ on HFD-induced obesity mouse model. The results showed that the administration of TFQ reduced body weight increasing and body lipid accumulation. TFQ gavage ameliorated the progression of fatty liver, as revealed by decreased hepatic triglyceride accumulation. TFQ improved insulin tolerance (lowered HOMA-IR index) and enhanced glucose utilization (decreased glucose AUC in OGTT), which may explain its preventive effects on visceral obesity and liver steatosis in part.

Under the influence of a high-fat diet, the changing of intestinal microbiota activates immune cells residing in the gut to release of inflammatory cytokine, which further impairs tight junction proteins and intestinal barrier function^29^. Consequently, LPS penetrates into the circulatory system and causes metabolic endotoxaemia, triggering obesity associated systemic inflammation and other metabolic disorders^28^. Consistent with these reports, our results indicated that HFD feeding activated NF-κB signaling pathway and elevated the expression of inflammatory cytokines (e.g., TNFα and COX-2), followed by impaired tight junction proteins and increased serum LPS penetration. TFQ treatment dramatically reduced the serum concentration of LPS induced by HFD feeding. The underlying mechanism might be multiplex. On one hand, flavonoids are reported to effectively suppress the TLR4/NF-κB signaling pathway and the subsequent pro-inflammatory cytokine expression^30^. On the other hand, flavonoids regulate the expression and assembly of tight junction proteins by influencing tyrosine kinases and PKCδ^31^. According to our observation, the flavonoid-rich TFQ could exert its effect on intestinal inflammation and intestinal barrier integrity in HFD-induced obesity mouse model via both of the regulatory pathways mentioned above.

Several lines of evidence suggest that gut microbiota play an important role in the development of obesity-associated pathologies^5,6^. Greater diversity of the intestinal microbiota appears to be negatively correlated with abnormal weight gain and type 2 diabetes^8,9^. In this study, TFQ supplementation significantly elevated alpha diversity and reshaped the structure of gut microbiota. Previous survey showed obese people had about 20% more *Firmicutes* and 90% less *Bacteroidetes* than lean people^32^. *Firmicutes* metabolized sugars more efficiently than *Bacteroidetes*, which was in favor of energy resorption and obesity. The higher ratio of *Firmicutes* to *Bacteroidetes* thus means the probability to more calorie intake and overweight^33,34^. TFQ treatment prominently decreased the ratio of *Firmicutes* to *Bacteroidetes*, which might be another mechanism to explain the improvement of TFQ in HFD-induced obesity and insulin resistance.

At the genus level, the HFD significantly reduced the number of *Akkermansia* and *Alistipes*. However, TFQ supplement recovered these beneficial bacteria. *Akkermansia* is a mucin-degrading bacterium of the phylum *Verrucomicrobia* commonly found in human gut^35^. Nowadays, it is accepted that *Akkermansia* is a kind of probiotics and the reduction of gut *Akkermansia* is associated with obesity related metabolic syndrome, albeit the exact mechanisms have not been fully elucidated. According to the literatures, *Akkermansia* using mucins as energy source stimulates goblet cells to produce mucus, which enhances mucus layer thickness and intestinal barrier^36^. From this point of view, TFQ stimulated increase in *Akkermansia* population might contribute to the reduction of gut permeability and LPS leakage. Studies have shown that many flavonoid-rich substances such as cranberry extract and green tea leaves are also able to increase the proportion of *Akkermansia* in animal or human gut^27,37^. *Alistipes* is a *Bacteroidetes* member of the family *Rickenellaceae*. Studies have demonstrated a negative correlation between obesity and the abundance of *Alistipes* in the gut^38^. The increase of *Alistipes* in the gut of TFQ treated HFD-fed mice may aid in its obesity prevention effect. In addition, HFD increased the abundance of *Dubosiella*, *Faecalibaculum* and *Lactobacillus*, while TFQ treatment normalized these bacteria close to the level of chow-fed group. Although the effects of these bacteria on lipid and glucose metabolism are not well-documented, those might be conducive to keep the energy homeostasis of the body as well. Therefore, we conclude TFQ is beneficial to the balance of glycolipid metabolism and prevention of obesity probably through restoration of specific flora to a normal healthy baseline.

In summary, TFQ prevents HFD-induced obesity, insulin resistance and liver steatosis. These effects are related to the upregulation of tight junction proteins and the reduction of metabolic endotoxemia. Our study further suggests that TFQ could modulate gut microbiota by reducing the ratio of *Firmicutes* to *Bacteroidetes*, and modulating the relative abundance of some genera, including *Akkermansia*, *Alistipes*, *Dubosiella*, *Faecalibaculum*, and *Lactobacillus*. These findings support a prebiotic role of TFQ as a dietary supplement for the intervention of gut dysbiosis and obesity-related metabolic disorders.

## Supplementary information

Supplementary Figure Legends

Figure S1

Figure S2

Figure S3

Figure S4

Figure S5
