# Supplementary material for: The flavonoid-rich Quzhou Fructus Aurantii extract modulates gut microbiota and prevents obesity in high-fat diet-fed mice
Source: Nutr Diabetes. 2019 Oct 23;9:30. doi: 10.1038/s41387-019-0097-6 (PMC6811639; doi:10.1038/s41387-019-0097-6)
Supplement: Supplementary file 1 — Supplementary Figure Legends [file 41387_2019_97_MOESM1_ESM.docx]

**Supplementary Figure Legends**

**Supplemental Fig. S1 The effect of TFQ on obesity in HFD-fed mice. (A)** Total weight gain (n=12). **(B)** The representative images of H&E staining in epididymal white adipose tissue. Scale bar, 300 μm. Data were expressed as the mean ± SD. ^**^*p*<0.01, versus chow group; ^#^*p*<0.05, versus HFD group.

**Supplemental Fig. S2 The effect of TFQ on serum lipid levels in HFD-fed mice. (A, B)** Serum cholesterol (TC) and triglyceride (TG). **(C)** Serum high density lipoprotein cholesterol (HDL-C) level. **(D)** Serum low density lipoprotein cholesterol (LDL-C) level. **(E)** Serum Non-Esterified Fatty Acid (NEFA) level. Data were expressed as the mean ± SD (n=12). ^*^*p*<0.05, ^**^*p*<0.01, versus chow group; ^#^*p*<0.05, ^##^*p*<0.01, versus HFD group.

**Supplemental Fig. S3 The effect of TFQ on fatty liver in HFD-fed mice.** Serum alanine aminotransferase (ALT) and aspartate aminotransferase (AST) activities **(A)**, liver weight **(B)**, hepatic cholesterol (TC) and triglyceride (TG) levels (C) were assessed in mice. Data were expressed as the mean ± SD (n=12). ^*^*p*<0.05, ^**^*p*<0.01, versus chow group; ^#^*p*<0.05, ^##^*p*<0.01, versus HFD group.

**Supplemental Fig. S4 The effect of TFQ on insulin resistance in HFD-fed mice. (A)** Oral glucose tolerance test (OGTT, 2 g/kg body weight, p.o.) in each group mice at week 8. **(B)** Insulin tolerance test (ITT, 0.75 U/kg body weight, i.p.) in each group mice at week 7. Data were expressed as the mean ± SD (n=5). ^*^*p*<0.05, ^**^*p*<0.01, versus chow group; ^#^*p*<0.05, versus HFD group.
