# Supplementary figures and images for: The flavonoid-rich Quzhou Fructus Aurantii extract modulates gut microbiota and prevents obesity in high-fat diet-fed mice
Source: Nutr Diabetes. 2019 Oct 23;9:30. doi: 10.1038/s41387-019-0097-6 (PMC6811639; doi:10.1038/s41387-019-0097-6)

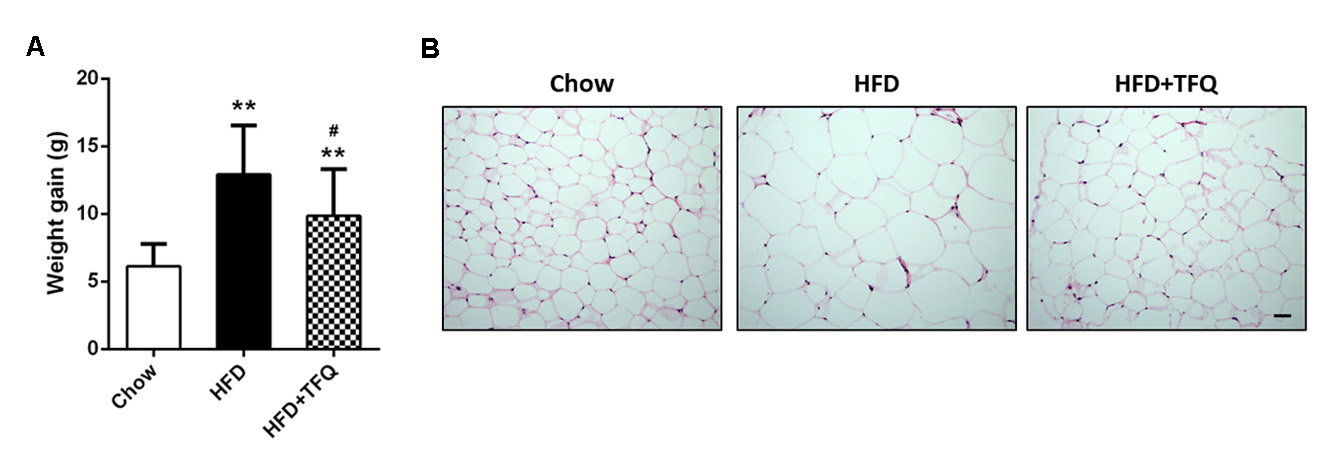

Supplement: Supplementary file 2 — Figure S1 [file 41387_2019_97_MOESM2_ESM.tif]

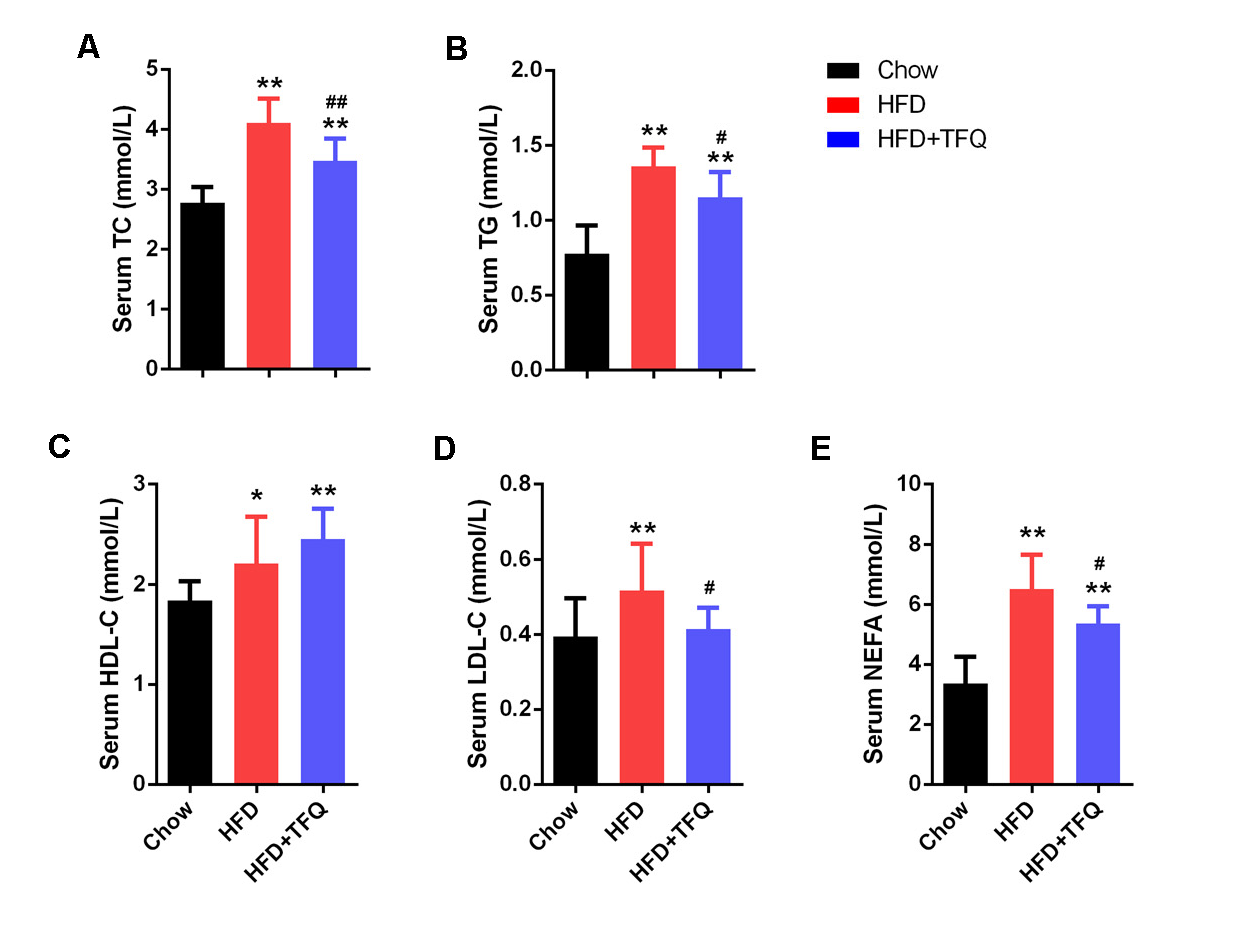

Supplement: Supplementary file 3 — Figure S2 [file 41387_2019_97_MOESM3_ESM.tif]

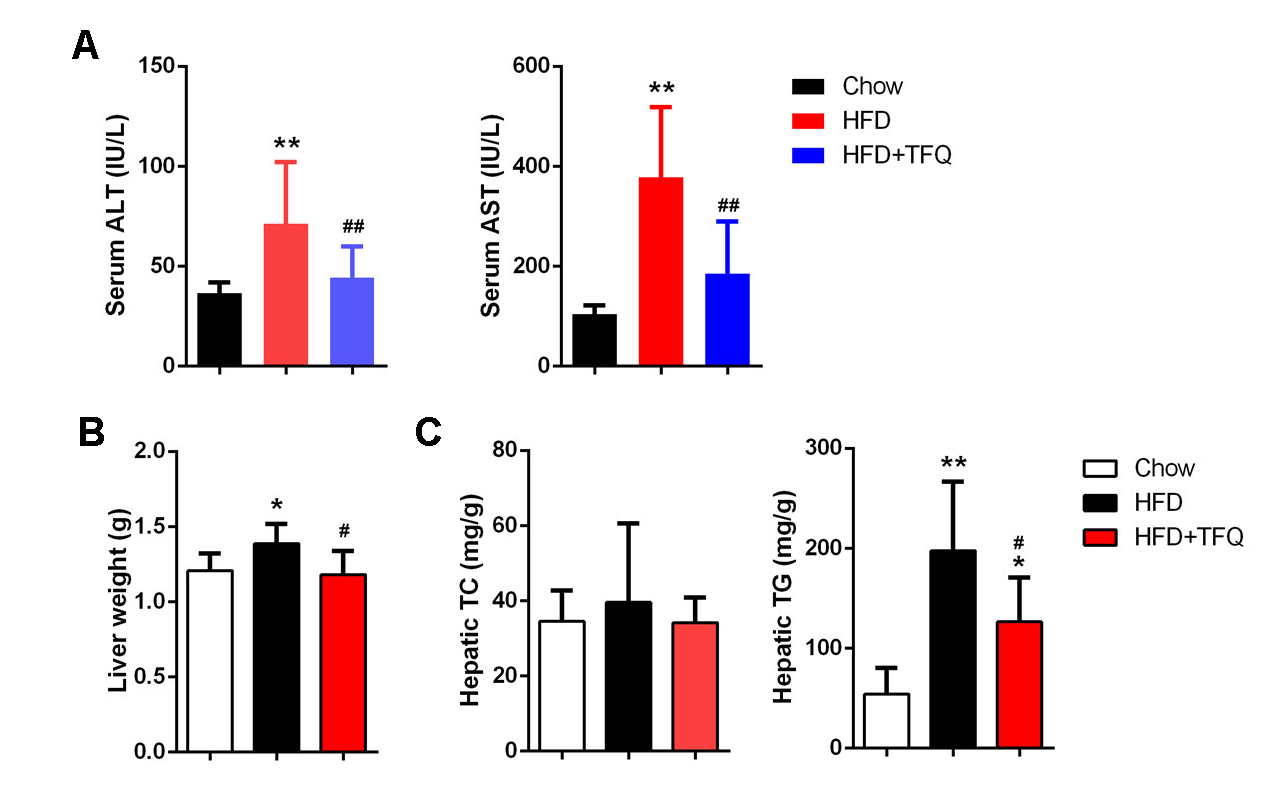

Supplement: Supplementary file 4 — Figure S3 [file 41387_2019_97_MOESM4_ESM.tif]

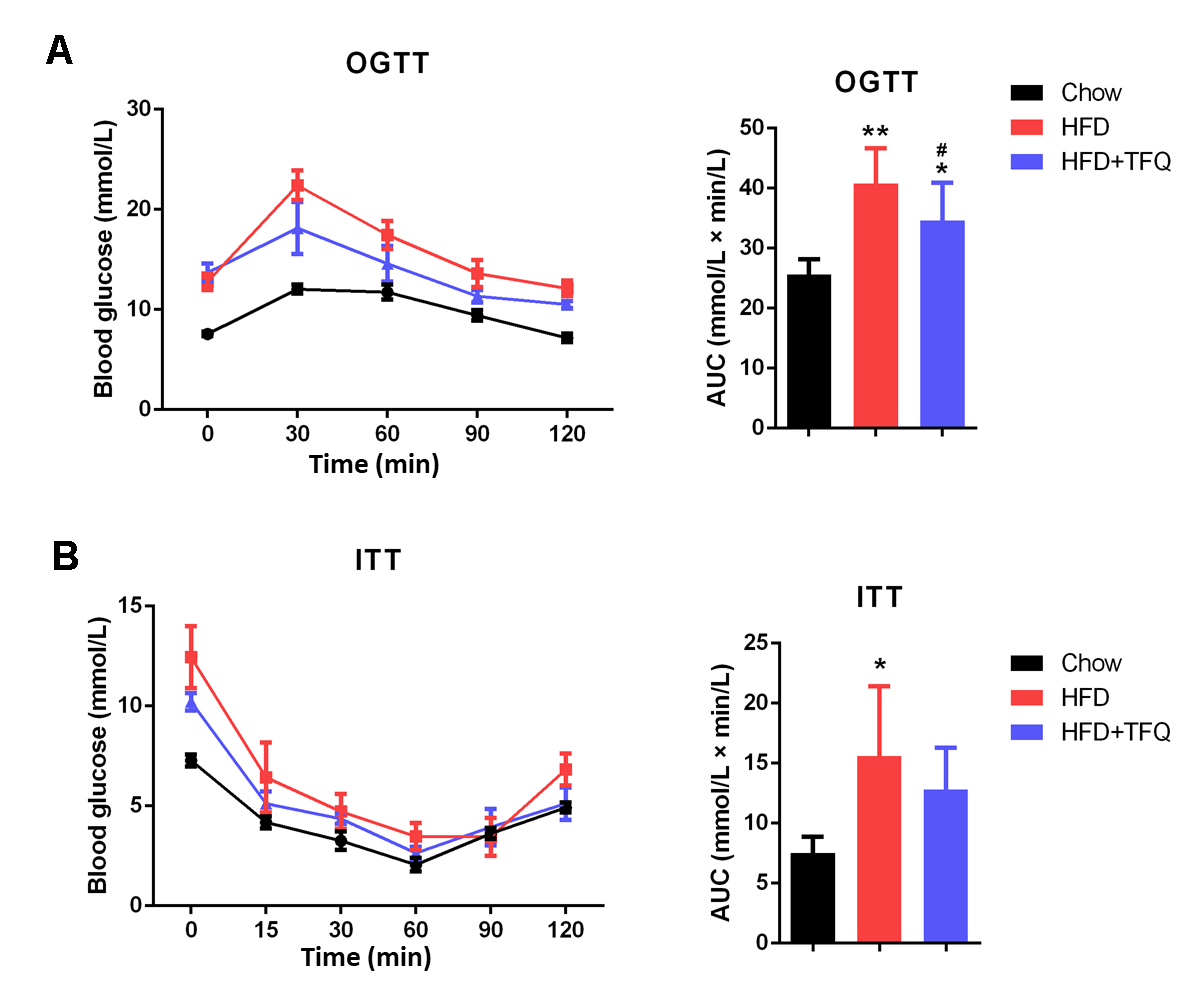

Supplement: Supplementary file 5 — Figure S4 [file 41387_2019_97_MOESM5_ESM.tif]

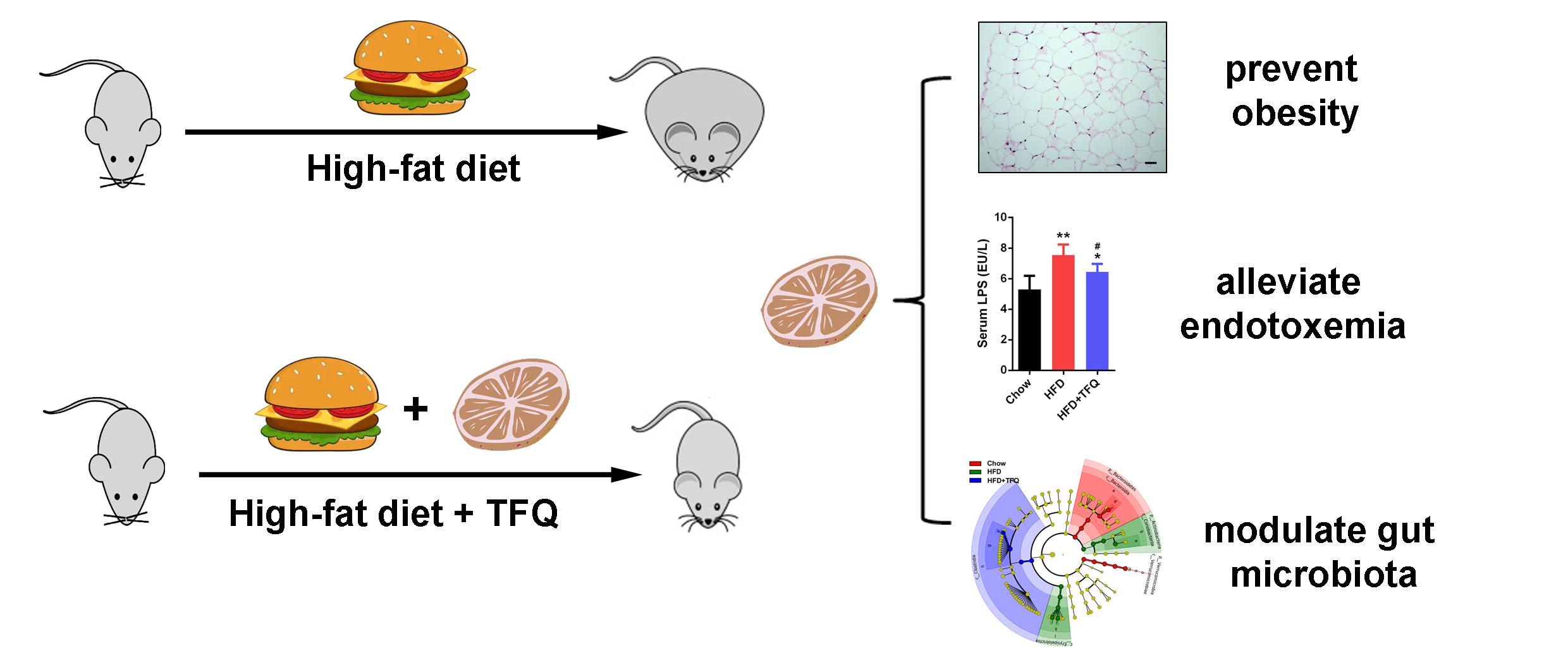

Supplement: Supplementary file 6 — Figure S5 [file 41387_2019_97_MOESM6_ESM.tif]
